# Targeting Mitochondrial Dynamics via EV Delivery in Regenerative Cardiology: Mechanistic and Therapeutic Perspectives

**DOI:** 10.3390/cells14211738

**Published:** 2025-11-05

**Authors:** Dhienda C. Shahannaz, Tadahisa Sugiura, Brandon E. Ferrell, Taizo Yoshida

**Affiliations:** 1 Faculty of Medicine, Universitas Indonesia, Jakarta 10430, Indonesia; dhiendaladdynasrul@gmail.com; 2 Department of Cardiothoracic and Vascular Surgery, Montefiore Medical Center/Albert Einstein College of Medicine, Bronx, NY 10467, USA; 3 Department of General Surgery, Montefiore Medical Center/Albert Einstein College of Medicine, Bronx, NY 10467, USA

**Keywords:** mitochondrial dynamics, extracellular vesicles (EVs), cardiac regeneration, heart failure therapy, mitochondrial transfer, regenerative cardiology, stem cell-derived EVs, mitochondrial biogenesis and mitophagy, EV-based drug delivery, translational cardiovascular medicine

## Abstract

Mitochondrial dysfunction is a key contributor to cardiac injury and heart failure, and extracellular vesicles (EVs) have emerged as promising therapeutic agents due to their ability to deliver mitochondrial-targeted cargo. This review systematically maps the evidence on how EVs modulate mitochondrial dynamics—including fusion, fission, mitophagy, and biogenesis—in regenerative cardiology. We comprehensively searched PubMed, Scopus, and Web of Science up to September 2025 for original studies. A total of 48 studies were included, with most utilizing EVs from mesenchymal stem cells, induced pluripotent stem cells, or cardiac progenitors. The review found that EV cargo influences key pathways such as DRP1 and MFN2, restores mitochondrial membrane potential, reduces ROS accumulation, and improves cardiomyocyte survival. While engineered EVs showed enhanced specificity, a lack of standardized preparation and quantitative assessment methods remains a significant challenge. We conclude that EV-mediated mitochondrial modulation is a promising strategy for cardiac repair, but the field needs harmonized protocols, deeper mechanistic understanding, and improved translational readiness to advance beyond preclinical research. The future of this research lies in integrating systems biology and precision targeting.

## 1. Introduction

Cardiovascular disease (CVD) remains the leading global cause of death, with specific conditions such as myocardial infarction, ischemia/reperfusion injury, and chronic heart failure accounting for substantial morbidity. In a cohort study, the incidence of these conditions was observed in 4% of individuals younger than 60 years and 10% of those aged 60 years or older [1]. Recovery of functional myocardium is limited because adult cardiomyocytes are largely post-mitotic. Moreover, prior stem cell therapies have shown limited long-term engraftment and functional benefit, highlighting the need for alternative strategies. Mitochondrial dysfunction—manifesting as impaired oxidative phosphorylation, excessive reactive oxygen species (ROS) generation, dysregulated calcium buffering, mtDNA damage, and disrupted mitochondrial biogenesis—plays a central role in post-injury cell death, adverse remodeling, and contractile decline [2,3]. Recent advances have illuminated molecular regulators of mitochondrial dynamics—fusion proteins MFN1/2, OPA1; fission regulator DRP1; mitophagy controllers PINK1/Parkin; and quality control via mitochondrial derived vesicles (MDVs) and mitocytosis—as potential therapeutic nodes [4,5,6,7].

Parallel progress in extracellular vesicle (EV) biology has revealed EVs (including small EVs/exosomes and large EVs/microvesicles) as vectors of intercellular communication. They carry proteins, RNAs/miRNAs, lipids, and in some settings mitochondrial components or even intact mitochondria [3]. These natural nanoscale carriers have shown capacity to modulate target cell function. Specifically, they influence mitochondrial membrane potential (ΔΨm), ROS clearance, mitochondrial biogenesis via PGC-1α, antioxidant enzyme induction, and rescue of electron transport chain components under stress. EV biogenesis pathways—via multivesicular bodies (MVBs), ESCRT machinery, Rab GTPases (for example Rab7), and autophagy/lysosomal crosstalk—dictate cargo loading, heterogeneity, and release dynamics [5,7,8,9].

### 1.1. Rationale

Evidence in recent preclinical models underscores that EVs derived from mesenchymal stem cells (MSCs), cardiac progenitors, iPSC-derived cardiomyocytes (iPSC-CMs), and other regenerative cell types [6,10,11] can attenuate ischemic and toxic injury by delivering mitochondrial-repair cargo. For example, EVs carrying ATP synthase subunits and electron-transport–complex proteins restore ATP production and improve oxidative phosphorylation (OCR) in hypoxic cardiomyocytes. EV-mediated activation of mitophagy via PINK1/Parkin also reduces accumulation of depolarized mitochondria and mitochondrial ROS during reperfusion injury [12,13,14]. iPSC-CM-derived EVs (including mitochondria-rich EVs or mitoEVs) have been shown to transfer mtDNA, TFAM, and OXPHOS components. These transfers transiently reconstitute ΔΨm, raise ATP/ADP ratios, and normalize calcium handling in injured cardiomyocytes, thereby limiting apoptotic signaling (BAX/BCL-2 balance) and caspase activation [2,12]. EVs modulate mitochondrial dynamics via regulators such as DRP1 and OPA1, and deliver miRNAs that fine-tune fusion/fission balance and mitophagy [13,15]. Despite promising functional readouts, substantial gaps persist: specificity of EV tropism to cardiomyocytes in vivo, quantitative assays for mitochondrial integration and long-term functional persistence of transferred organelles, the inflammatory risk posed by extracellular mtDNA/mitoDAMPs, and the urgent need for standardized EV isolation, characterization, dosing, and biodistribution protocols to enable reproducible translation [2,12,13,14,15].

### 1.2. Aim of the Review

This review synthesizes biomolecular mechanisms by which EVs modulate mitochondrial dynamics in cardiac regeneration, surveys recent therapeutic preclinical evidence, and identifies translational barriers and opportunities. By integrating insights from molecular biology, EV engineering, and cardiac pathophysiology, we outline how EV-based mitochondrial targeting may emerge as a natural evolution beyond traditional stem cell therapies, becoming a clinically viable strategy for myocardial repair.

## 2. Materials and Methods

To ensure methodological rigor and comprehensive coverage of the literature, a systematic search strategy was employed across multiple scientific databases, including PubMed, Scopus, Web of Science, and Embase, spanning the period 2006–2025. Search terms were selected to capture all relevant mechanistic and therapeutic studies involving mitochondrial dynamics and extracellular vesicle (EV) interventions in the context of cardiac regeneration. Core keywords included: “mitochondrial dynamics,” “mitochondrial fusion,” “mitochondrial fission,” “mitophagy,” “transmitophagy,” “biogenesis,” “extracellular vesicles,” “exosomes,” “microvesicles,” “cardiac regeneration,” “myocardial repair,” “EV delivery,” and their combinatorial Boolean variations.

Inclusion criteria encompassed original in vitro, ex vivo, and in vivo studies reporting EV-mediated modulation of mitochondrial function in cardiomyocytes, cardiac progenitors, or relevant cardiac injury models. Studies employing engineered EVs or modified cargo for targeting mitochondrial pathways were prioritized. Exclusion criteria included studies not addressing cardiac contexts, EVs unrelated to mitochondrial modulation, or publications lacking mechanistic data. Non-English articles were included if sufficient English abstracts and data were available.

The study selection process followed a structured three-step approach. First, titles and abstracts were screened independently by two reviewers to identify potentially eligible studies. Second, full texts were assessed for relevance and methodological detail, including EV characterization (size, surface markers, source cell type), cargo content (proteins, miRNAs, mtDNA, signaling molecules), and mitochondrial outcomes (fusion/fission balance, membrane potential, reactive oxygen species, bioenergetic indices). Third, discrepancies were resolved via consensus, ensuring high fidelity and reproducibility.

A data charting framework was developed to extract key biomolecular and experimental details systematically. Extracted parameters included: experimental model, EV source and isolation method, cargo composition, molecular targets (DRP1, MFN1/2, OPA1, PINK1/Parkin), mitochondrial functional outcomes, cardiomyocyte viability or contractility indices, and limitations. Quantitative data, where available, were summarized to allow cross-study comparisons and highlight mechanistic trends. Graphical representations were prepared to illustrate EV–mitochondria interactions and pathway modulation.

This structured, detail-oriented methodology emphasizes clarity, reproducibility, and translational relevance, providing a rigorous foundation for synthesizing current mechanistic insights and therapeutic applications of EV-mediated mitochondrial modulation in regenerative cardiology.

## 3. Conceptual Framework

**Figure 1:**
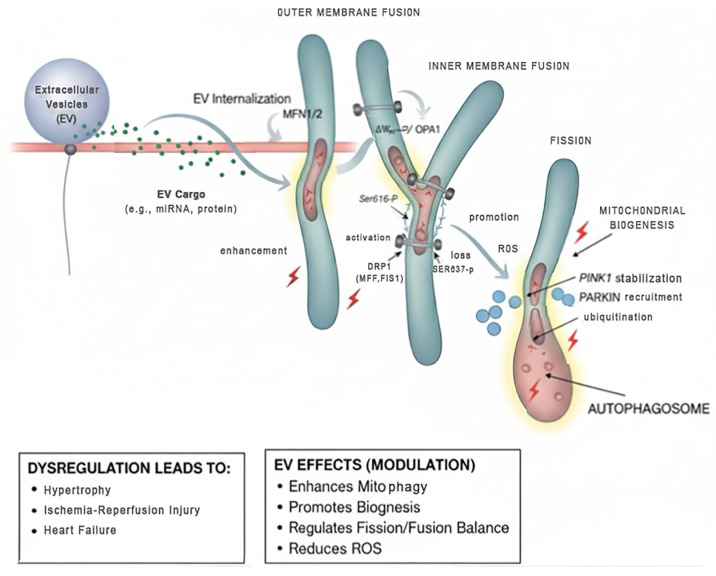
Conceptual EV–Mitochondrial Interaction in Cardiomyocytes. EV: Extracellular Vesicle; microRNAs: Micro Ribonucleic Acids (small non-coding RNA molecules); EV Internalization: The process by which Extracellular Vesicles are taken into the cell; MFN1/2: Mitofusin 1 and Mitofusin 2 (proteins involved in outer mitochondrial membrane fusion); ΔΨm: Mitochondrial membrane potential; OPA1: Optic Atrophy 1 (a protein involved in inner mitochondrial membrane fusion); DRP1: Dynamin-Related Protein 1 (a protein involved in mitochondrial fission); MFF: Mitochondrial Fission Factor (a protein involved in recruiting DRP1 to the mitochondria); FIS1: Fission 1 (a protein involved in mitochondrial fission); Ser616-P: Serine 616 Phosphorylation (a modification that activates DRP1); Ser637-P: Serine 637 Phosphorylation (a modification that inactivates/leads to loss of DRP1 activity); ROS: Reactive Oxygen Species; PINK1: PTEN-induced kinase 1 (a mitochondrial kinase); PARKIN: Parkin RBR E3 ubiquitin protein ligase (an E3 ubiquitin ligase). Color meanings: Light Blue/Gray (Outer Membrane and Inner Membrane Structures): Represents the mitochondrial membranes and the overall structure of mitochondria. The light blue is the outer membrane, and the slightly darker blue is the inner membrane. Green Spheres (within EV and entering cell): Represent the “EV Cargo” (e.g., microRNAs, proteins) being delivered by the extracellular vesicles. Small Red Spheres/Dots (ROS): Indicate Reactive Oxygen Species, often associated with cellular stress or damage. Darker Blue Spheres (within Autophagosome): Represent components or damaged parts of mitochondria being targeted for degradation. Pink/Red (Lightning bolt symbols and shading): This color, especially the lightning bolts, generally signifies “Enhancement” or “Promotion” of a process. It might also subtly indicate energy or a critical regulatory step. The red shading within the autophagosome signifies the degradation process. Arrows and connecting lines: Solid Black Arrows (Unidirectional): Indicate the direction of a process or a flow of material; Example: EV to EV Internalization, EV Internalization to Mitochondria. Example: Fission leading to smaller mitochondrial fragments and eventually mitophagy. Bidirectional Arrows (e.g., between MFN1/2 and mitochondrial outer membrane) suggest an interaction, binding, or a dynamic equilibrium. Arrows pointing to lightning bolts indicate that the process or molecule is “Enhanced” or “Promoted” by the preceding step. Lines connecting abbreviations to structures show where these proteins or factors are acting; for example, MFN1/2 acting on the outer membrane fusion and DRP1, MFF, FIS1 acting on mitochondrial fission points. Arrows showing “Activation” or “Loss” specifically highlight the functional consequence of phosphorylation (e.g., Ser616-P leads to activation).

Mitochondrial dynamics—balanced cycles of fusion, fission, biogenesis and selective removal (mitophagy)—are central to cardiomyocyte bioenergetics and stress resilience. Outer-membrane fusion is mediated by mitofusins MFN1/2, inner-membrane fusion by OPA1; fission is executed by DRP1, whose activity is regulated by site-specific phosphorylation (e.g., Ser616 activation vs. Ser637 inhibitory phosphorylation) and receptor adaptors (MFF, FIS1) that recruit DRP1 to the outer mitochondrial membrane. Impaired coordination of these processes triggers mitochondrial fragmentation, loss of membrane potential (ΔΨm), excessive ROS, and activation of PINK1 stabilization on depolarized mitochondria with downstream Parkin recruitment—leading to ubiquitination and autophagic clearance; dysregulation of any node contributes to hypertrophy, ischemia–reperfusion injury and heart failure [10,16,17,18,19], as illustrated in Figure 1.

### 3.1. Mitochondrial Remodeling in Cardiac Stress and Repair

Beyond canonical dynamics, mitochondria undergo structural and metabolic remodeling that critically shapes cardiomyocyte adaptation to stress. Remodeling encompasses cristae reshaping, supercomplex assembly, and shifts in substrate utilization, collectively optimizing oxidative phosphorylation efficiency. Inner-membrane fusion protein OPA1 mediates cristae architecture [20], while the MICOS complex regulates cristae junction stability and electron transport chain (ETC) supercomplex organization [21]. EVs, particularly from iPSC-derived cardiomyocytes (iPSC-CMs), can promote metabolic maturation by enhancing oxidative metabolism over glycolysis, likely via delivery of proteins and miRNAs that fine-tune ETC assembly and mitochondrial substrate preference. Integrating remodeling into the EV–mitochondrial axis expands the mechanistic framework: EVs not only restore structural integrity and dynamics but also recalibrate metabolic and bioenergetic competence in stressed cardiomyocytes [21].

### 3.2. Mitochondrial Remodeling + Systems Medicine

Structural remodeling of mitochondria not only restores organelle integrity but also drives functional adaptation across the cardiac cellular ecosystem. EV-mediated interventions can recalibrate cardiomyocyte metabolism, promote substrate flexibility, and enhance immunometabolic crosstalk with resident fibroblasts and immune cells. By integrating remodeling with systems-level considerations, EVs act as modulators of networked bioenergetics, coordinating oxidative phosphorylation, ROS buffering, and inflammatory signaling to optimize cardiac repair. This perspective frames remodeling as an active driver of therapeutic benefit rather than a mechanistic footnote, highlighting the interplay between structural dynamics, metabolic reprogramming, and multicellular functional integration.

EVs—including small exosomes (50–150 nm), larger microvesicles, and mitochondrial-derived vesicles (MDVs)—carry complex cargo (mtDNA, mitochondrial proteins such as TFAM and components of the electron transport chain, cardiolipin, mitochondrial RNAs, miRNAs and proteins that regulate fission/fusion) and can affect intercellular modulation of mitochondrial quality control. EVs may deliver intact mitochondrial fragments or functional mitochondria (mitoEVs/M-EVs) that transiently restore respiration, or transfer regulatory small RNAs that alter expression or post-translational modification of DRP1, MFN2 or OPA1 in recipient cardiomyocytes [7,22].

Mechanistically, EV uptake by cardiomyocytes occurs via endocytosis, membrane fusion or receptor-mediated internalization; subsequent release of mitochondrial cargo into the cytosol or direct mitochondrial fusion can replenish ATP production and reduce ROS, while EV-delivered miRNAs can downregulate pro-fission signaling (e.g., suppress DRP1 expression) or upregulate mitofusins and biogenesis programs (PGC-1α pathway). Preclinical studies demonstrate that MSC-derived and iPSC-derived EVs stabilize mtDNA, improve ΔΨm, reduce ROS and activate PINK1/Parkin-dependent quality control—effects amplified when EVs are engineered for mitochondrial targeting or loaded with specific miRNAs/proteins [23,24,25]. Key regulators are summarized in Table 1.

In addition to revitalization, extracellular vesicles play an emerging role in mitochondrial quality control through a process known as transmitophagy—the intercellular transfer and degradation of damaged mitochondria. This mechanism complements traditional mitophagy by allowing cells to outsource mitochondrial clearance via vesicular pathways, thereby preserving tissue homeostasis under stress conditions [17]. Integrating transmitophagy into the framework of mitochondrial EV biology highlights a broader function of EVs beyond metabolic rescue—extending to quality control and mitochondrial turnover across tissues.

## 4. Current Landscape: What Has Been Done

Preclinical studies over the last decade demonstrate progressively convincing evidence that extracellular vesicles (EVs) can modulate cardiomyocyte mitochondrial function via delivery of proteins, RNAs, and intact mitochondrial components [2,3]. Early mechanistic work showed that mesenchymal stem cells (MSCs) offload damaged mitochondria via microvesicle-like structures that are then processed by recipient phagocytes to influence tissue bioenergetics, establishing a biological precedent for EV-mediated mitochondrial quality control [33].

### 4.1. iPSC-CM Derived EVs: Bridging Stem Cell Therapy and Mitochondrial Transfer

iPSC-CM-derived EVs represent a convergence of stem cell therapy and organelle-level mitochondrial transfer, offering a dual modality for myocardial repair. Beyond providing paracrine signals that enhance cardiomyocyte maturation and survival, these EVs deliver functional mitochondrial components, including ETC proteins, mtDNA, and metabolically active mitochondria, thereby complementing canonical metabolic maturation strategies in iPSC-CMs [2,3]. Notably, our work demonstrates that iPSC-CM EVs can accelerate oxidative metabolism, modulate fusion/fission dynamics, and rescue ΔΨm in stressed cardiomyocytes, filling a critical gap between conventional iPSC-derived cell therapy and direct mitochondrial replacement approaches. These findings suggest a translationally tractable route for precision EV therapy that leverages both network-level signaling and direct bioenergetic restoration, establishing iPSC-CM EVs as a mechanistic bridge from preclinical studies to potential clinical interventions [12,13,14,15]. These findings [2,3,12,13,14,15], led by our group, establish a foundational mechanistic framework that uniquely positions iPSC-CM EVs as a translational bridge between preclinical studies and clinical applications.

### 4.2. EV Sources and Cargo

Most cardiac-focused studies use EVs derived from MSCs, induced pluripotent stem cell (iPSC)-derived cardiomyocytes, cardiac progenitor cells, or cardiac fibroblasts. MSC-EVs commonly carry mitochondria-protective microRNAs (e.g., miR-21, miR-222), mitochondrial proteins (ATP synthase subunits), and mitochondrial DNA fragments that correlate with preserved oxidative phosphorylation (OXPHOS) and reduced ROS in recipient cardiomyocytes [24,34]. Notably, “mito-EVs”—EVs containing intact or partial mitochondria—have been isolated from iPSC–cardiomyocyte cultures and shown to restore mitochondrial respiration in injured cardiomyocytes in vitro and improve function in large-animal MI models [24,35].

### 4.3. Delivery Methods and Tissue Targeting

Studies have employed intravenous, intracoronary, and intramyocardial delivery routes; larger mitochondria-rich EVs (L-EVs) appear more likely to fuse directly with cardiomyocyte membranes, whereas small EVs (sEVs/exosomes) act via endocytosis and cargo release into endolysosomal or cytosolic compartments. Cargo engineering (miRNA loading, peptide-targeting moieties, or surface display of cardiotropic ligands) has improved myocardial uptake and mitochondrial targeting in rodent models [36,37].

### 4.4. Impact on Mitochondrial Dynamics and Downstream Physiology

Mechanistic readouts consistently report modulation of canonical mitochondrial regulators: decreased DRP1 phosphorylation (reduced fission), increased MFN1/MFN2/OPA1 expression (enhanced fusion), activation of PINK1–Parkin-mediated mitophagy where appropriate, restoration of mitochondrial membrane potential (ΔΨm), improved complex I–IV respiration, and lowered mitochondrial ROS. These molecular shifts translate to improved ATP content, preserved calcium buffering, reduced cardiomyocyte apoptosis, and smaller infarct sizes in ischemia–reperfusion and chronic heart-failure models [7,10].

### 4.5. Mechanistic Pathways Summarized

EV cargo exerts effects through: (1) direct mitochondrial donation/fusion, (2) delivery of regulatory miRNAs that downregulate fission mediators or upregulate fusion/biogenesis genes (PGC-1α, NRF1), (3) transfer of mitochondrial proteins that stabilize electron transport chain complexes, and (4) modulating recipient cell mitophagy and inflammatory signaling. Recent omics and imaging studies corroborate functional mitochondrial transfer and bioenergetic rescue [5,38].

### 4.6. Clinical Relevance and Limitations

Translation is nascent: reproducible large-animal efficacy exists (porcine MI models) but human trials specifically examining mito-targeting EVs are limited; safety, immunogenicity, and standardized manufacturing remain unresolved. Heterogeneity in EV isolation, inconsistent dosing metrics (particle vs. protein vs. functional titers), and inadequate in vivo tracking impede cross-study comparability. Key gaps include quantitative assays for mitochondrial transfer, long-term fate of donor mitochondria, and off-target effects (immune activation, oncogenic risk) [39,40].

### 4.7. Clinical Trials and Translational Landscape

The translation of EV-based mitochondrial therapies from preclinical models into human studies is accelerating but remains at an early phase. Systematic evaluations of registered clinical trials indicate an increasing number of EV-related interventional studies across therapeutic areas, and early-phase cardiac-focused trials have begun to appear. Notably, a contemporary systematic review of EV clinical trials documented a rapid expansion of EV interventional studies and highlights the predominance of phase I/II designs focused on safety and feasibility. In the diovascular domain specifically, several early-phase and first-in-human studies now evaluate MSC- or stem cell-derived EVs administered by intravenous or intracoronary routes; at least one protocol evaluating intravenous MSC-derived EVs in cardiac indications is registered (ClinicalTrials.gov NCT06002841 [41]), signifying a move from preclinical large-animal validation toward human testing. These trials are typically designed to address (a) safety and immunogenicity, (b) biodistribution and persistence, and (c) early efficacy signals (cardiac biomarkers, imaging endpoints). These endpoints reflect the critical translational questions for mitochondrial-targeted EVs: whether mitochondrial cargo (mtDNA, TFAM, ETC proteins, intact mitochondria) persists, integrates functionally into host mitochondria, and improves organ-level bioenergetics without provoking detrimental inflammatory responses to extracellular mitochondrial components (e.g., mitoDAMPs).

Table 2 summarizes representative preclinical and early clinical studies that directly evaluated EV-mediated mitochondrial outcomes in cardiac contexts or that are registered to test EV therapeutics in cardiac patients. The table highlights key mechanistic endpoints (ΔΨm, ATP production, OCR, PINK1/Parkin activation), delivery routes, and principal translational limitations.

### 4.8. Regulatory and Manufacturing Challenges

The principal translational bottlenecks for EV-based mitochondrial therapies are regulatory classification, potency assays, and scalable GMP manufacture. Regulatory agencies (FDA/EMA) currently consider EV therapeutics in contexts that span biologics, cell-derived products, and drug-delivery platforms; this creates ambiguity for potency definitions and comparability criteria. EVs carrying mitochondrial cargo raise unique safety concerns (extracellular mtDNA and mitoDAMPs) that can elicit innate immune activation via cGAS–STING/TLR9 pathways; thus, preclinical packages must include sensitive assays for immune activation and for potential horizontal transfer of mtDNA variants.

From a manufacturing standpoint, scalable production of mitochondria-enriched EV subpopulations (mito-EVs/L-EVs) remains technically demanding. Standardization requires (i) source cell banking under GMP, (ii) scalable culture systems (bioreactors), (iii) robust fractionation (TFF + SEC) to separate small EVs from L-EV/mito-EV fractions, and (iv) validated potency assays tailored to mitochondrial rescue (e.g., donor EV ATP-generating capacity, recipient ΔΨm rescue potency, or OCR-based functional assays). Recent progress in tangential flow filtration and multi-omic EV characterization provide feasible paths forward, but consensus potency endpoints remain to be defined.

### 4.9. Synthesis

Collectively, preclinical evidence supports EV-mediated modulation of mitochondrial dynamics as a mechanistically plausible and therapeutically promising strategy for myocardial repair. Rapid progress in engineered EVs and improved characterization methods make clinical translation feasible—provided that rigorous standardization, biodistribution profiling, and safety studies are prioritized [36,46].

## 5. Emerging Trends and Innovative Angles

This section synthesizes the most actionable, biomolecularly detailed innovations in EV-based mitochondrial targeting for cardiac repair over the past two decades, emphasizing translationally tractable strategies and experimental evidence. Each subsection highlights preclinical versus early clinical readiness where applicable.

### 5.1. Engineered EVs for Targeted Mitochondrial Modulation (Cargo Design + Targeting Motifs)

Engineered EVs are routinely loaded with mitochondria-protective proteins (e.g., SIRT3, ATP5A1), mitochondria-stabilizing small proteins/peptides, mito-protective miRNAs, and enzymatic antioxidants. These cargos directly restore bioenergetics and redox balance in injured cardiomyocytes. Recent reports demonstrate nanoscale exosomes co-loaded with SIRT3 and metabolic modulators that increase mitochondrial respiration (OCR), raise complex I–IV activity, and reduce mitochondrial ROS and mPTP opening after ischemia–reperfusion injury. Engineered surface ligands (peptide motifs targeting cardiomyocyte troponin I or ischemia-exposed integrins) enhance myocardial tropism and EV uptake via receptor-mediated endocytosis or fusion, increasing intramitochondrial rescue efficiency [10,47]. Translational status: mostly preclinical; cardiac-specific tropism strategies moving toward early large-animal studies.

### 5.2. Direct Mitochondrial Transfer and Mito-EVs (Large EVs and Mitochondrial-Containing EV Populations)

Larger EV subtypes (mitoEVs, M-EVs) can carry entire mitochondria or mitochondrial fragments (mtDNA, OXPHOS complexes). Transfer of these cargoes restores ATP production, ΔΨm, and respiratory chain integrity in recipient cardiomyocytes. Preclinical large-animal models show autologous M-EVs reduce infarct size and improve ejection fraction by reconstituting respiratory complexes and increasing ATP/ADP ratios in peri-infarct tissue. Mechanistically, transferred mitochondria integrate into host mitochondrial networks and promote fusion/fission recalibration via MFN2/OPA1 upregulation and DRP1 modulation [24,35]. Translational status: advanced preclinical; autologous human M-EV studies planned.

### 5.3. RNA-Based Mitochondrial Modulation (miRNA, gRNA, and Mitochondrial Delivery Routes)

EVs shuttle miRNAs that regulate key dynamics regulators (e.g., miR-499/-30 family affecting DRP1 phosphorylation, miR-21/-214 influencing PINK1/Parkin signaling). Recent work demonstrates EV-mediated mitochondrial import of small RNAs, enabling functional modulation of fusion/fission proteins and downstream mitophagy/apoptosis pathways (caspase-9, BAX/BCL2 balance). High-throughput barcoding of sEVs loaded with gRNA allows pooled screening of EV cargo effects on recipient mitochondrial phenotypes [48,49]. Translational status: preclinical; mechanistic proof-of-concept established.

### 5.4. Genome Editing and Programmable EV Payloads (CRISPR/Cas Systems in EVs)

EVs have been engineered to deliver CRISPR/Cas RNPs and base editors targeting nuclear-encoded mitochondrial regulators (e.g., DRP1 phosphorylation sites, MFN2 expression) or nuclear genes that indirectly regulate mitochondrial quality control. Encapsulation strategies protect RNPs during circulation and enable endosomal escape in cardiomyocytes. These approaches have been validated in noncardiac models and are now adapted for cardiac mitochondrial targets [50,51]. Translational status: early-stage preclinical.

### 5.5. Cross-Tissue Lessons and Systems Integration (Oncology, Neurology, Aging)

EV-mediated mitochondrial signaling in oncology and neurodegeneration governs metabolic rewiring, immune-metabolic crosstalk, and senescence. These insights are now repurposed for heart failure: EV-delivered mito-signals modulate immune cell phenotype (macrophage polarization), influence fibroblast activation, and alter cardiomyocyte substrate preference (fatty acid vs. glucose oxidation). Integration with immunometabolism enables combination strategies (EV + immunomodulator) to foster reparative inflammation resolution [5,11].

### 5.6. Big-Picture Synthesis: Systems-Level Integration of EV–Mitochondrial Therapies

While current studies elucidate detailed molecular mechanisms of EV-mediated mitochondrial modulation, a systems-level framework is needed to unify these insights and guide translational impact [52]. EVs should be conceptualized not merely as cargo carriers but as network modulators orchestrating bioenergetics across multiple cardiac cell types. Integration of metabolomics (ATP/ADP ratio, ROS flux, NAD+/NADH balance), single-cell and spatial omics (differential EV responses in cardiomyocytes, fibroblasts, immune cells), and network modeling offers a holistic understanding of EV-mediated cardiac repair [52,53]. This framework positions EVs as precision tools in systems medicine, linking mitochondrial bioenergetics, immunometabolism, and tissue remodeling to create a unifying paradigm for regenerative cardiology. Multi-omic integration can guide EV engineering, dosing, and patient-specific therapeutics, accelerating clinical translation [54,55,56].

Schematic illustration of how mitochondria-enriched extracellular vesicles (EVs) restore cardiomyocyte function. The figure summarizes the major EV subtypes (small EVs, large EVs, and mitochondria-derived EVs), their representative cargos, and the downstream remodeling effects that enhance mitochondrial structure, oxidative phosphorylation efficiency, and overall cardiac repair.

**Figure 2:**
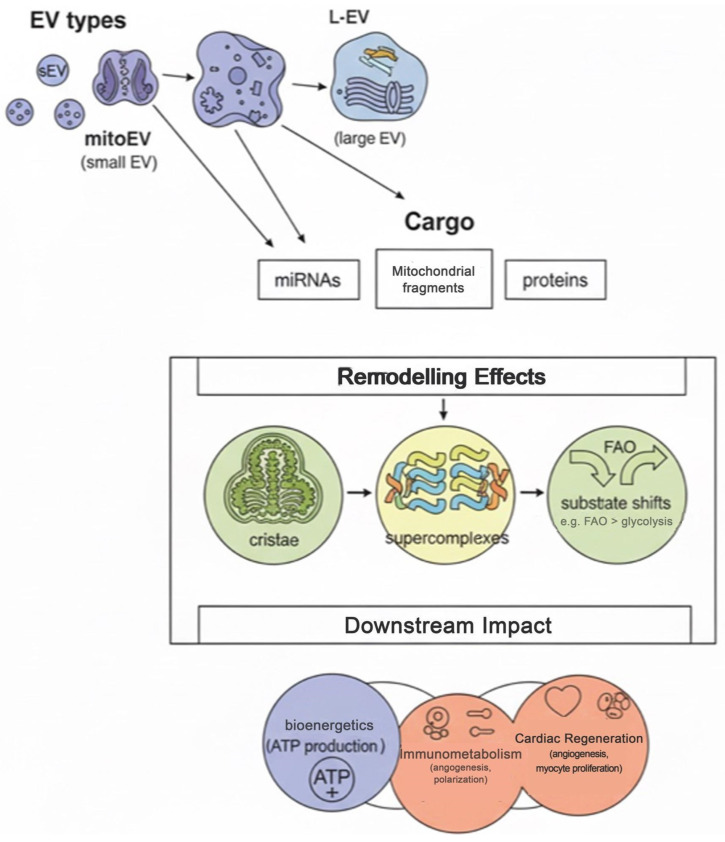
New Paradigm for EV–Mitochondrial Therapy.

Extracellular vesicles represent a multiscale mitochondrial delivery system encompassing small EVs (exosomes), large EVs (microvesicles), and mitochondria-derived EVs released under cellular stress (Figure 2). Their cargos—ranging from miRNAs and mitochondrial DNA to respiratory-chain proteins—collectively mediate mitochondrial repair and bioenergetic recovery in recipient cells. Following uptake, recipient mitochondria undergo cristae remodeling, stabilization of respiratory supercomplexes, and metabolic reprogramming that optimize oxidative phosphorylation and ATP generation. These remodeling events translate into improved cellular bioenergetics, reduced oxidative stress, and enhanced cardiomyocyte survival.

1. EV Types (The Delivery System): The figure depicts three primary types of EVs involved in mitochondrial communication and therapy: a. sEV (small EV): Generally referred to as exosomes, these are typically 30–150 nm in size. They are critical carriers of soluble factors and genetic material (miRNAs, proteins) that regulate mitochondrial function remotely, b. L-EV (Large EV): Also known as microvesicles or microparticles, these are larger (100–1000 nm+) and can carry entire organelle fragments, making them capable of transferring substantial mitochondrial components, c. mitoEV (Mitochondria-derived EV): A specialized subset, often released under cellular stress, that is specifically enriched with mitochondrial components (lipids, proteins, mtDNA fragments), acting as a highly targeted repair vehicle for damaged mitochondria. 2. Cargo (The Therapeutic Payload): These EVs carry a variety of molecular signals that mediate the therapeutic effect upon internalization by recipient cells: a. miRNAs (microRNAs): Non-coding RNAs that act as master regulators by targeting mRNA transcripts essential for mitochondrial biogenesis, dynamics, and the expression of Electron Transport Chain (ETC) subunits. b. Mitochondrial Fragments (Mitochondrial DNA/Lipids): Direct structural and functional components of the mitochondria that can be integrated into the recipient cell’s existing mitochondrial pool, providing raw materials for repair and replenishment of damaged organelles, c. Proteins: These include enzymes, transcription factors, and key components of the ETC (e.g., Complex I or III subunits) that are directly incorporated into the recipient cell’s mitochondria to immediately enhance or stabilize function. 3. Remodeling Effects (Recipient Mitochondrial Changes): Upon cargo delivery, the recipient cell’s mitochondria undergo structural and functional reorganization: a. Cristae: Remodeling of the inner mitochondrial membrane structure, often resulting in an increase in cristae density and complexity, which directly correlates with enhanced surface area for oxidative phosphorylation (OXPHOS) and increased ATP production capacity, b. Supercomplexes: The stable assembly and stabilization of respiratory supercomplexes (e.g., the Respirasome, involving Complexes I, III, and IV). This organizational structure optimizes electron transfer efficiency, reduces electron leakage, and minimizes the production of reactive oxygen species (ROS), c. Substrate Shifts (e.g., FAO, Glycolysis): Metabolic reprogramming where the cell’s preference for energy substrate changes. This can involve an improved capacity for Fatty Acid Oxidation (FAO), a shift toward optimized OXPHOS, or a switch toward glycolysis (Warburg effect) based on the specific tissue context and injury state. 4. Downstream Impact (Therapeutic Outcomes): The successful remodeling of recipient mitochondria translates into systemic and tissue-specific therapeutic benefits: a. Bioenergetics (ATP Production): The fundamental outcome is the restoration of cellular energy homeostasis, characterized by increased total ATP production, higher mitochondrial membrane potential (∆ψ m), and overall improved cell viability, b. Immunometabolism (Polarization): The influence of mitochondrial state on immune cell function. EV therapy can shift inflammatory immune cells (e.g., M1 macrophages) toward a reparative, anti-inflammatory phenotype (e.g., M2 polarization), modulating the local inflammatory environment, c. Cardiac Regeneration (Angiogenesis, Myocyte Proliferation): Specific to cardiovascular repair, the improved bioenergetics supports crucial healing processes, including stimulating angiogenesis (new blood vessel formation) and promoting the survival and proliferation of recipient cardiomyocytes (myocyte proliferation), leading to improved tissue function after injury.

### 5.7. Practical Translational Barriers and Technical Innovations

Key hurdles remain: EV heterogeneity, inconsistent isolation methods (ultracentrifugation, size-exclusion, tangential flow filtration), lack of quantitative metrics for mitochondrial cargo per vesicle, and incomplete biodistribution data. Emerging solutions include standardized multi-omic EV characterization workflows, CRISPR barcoding for in vivo tracking, and cardiomyocyte-targeted ligands to improve therapeutic index. Harmonized preclinical models and agreed reporting standards are essential to accelerate safe clinical translation [41,57].

### 5.8. Challenges and Knowledge Gaps in EV-Mediated Mitochondrial Modulation for Cardiac Regeneration

Despite the promising potential of extracellular vesicles (EVs) in targeting mitochondrial dynamics for cardiac repair, several technical, biological, and ethical challenges impede their clinical translation.

### 5.9. Technical Limitations

The isolation and characterization of EVs remain inconsistent due to the lack of standardized protocols, leading to variability in purity and yield. This heterogeneity complicates the assessment of their mitochondrial targeting efficiency and therapeutic efficacy. Moreover, tracking EVs in vivo poses significant challenges; current labeling techniques often suffer from low sensitivity and potential interference with EV function, hindering accurate biodistribution studies. Additionally, quantifying the precise mitochondrial effects of EVs is difficult due to the complex interplay of signaling pathways and the dynamic nature of mitochondrial processes. Advanced imaging and biochemical assays are required to dissect these interactions at a molecular level.

### 5.10. Biological Complexities

The specificity of EV-mediated delivery to target mitochondria is influenced by the source of EVs, their cargo content, and surface markers. While engineered EVs show promise in enhancing targeting precision, their design must consider the complex extracellular environment and potential interactions with non-target cells. Furthermore, the biodistribution of EVs is affected by systemic clearance mechanisms, including uptake by the mononuclear phagocyte system, which can limit their therapeutic reach. Understanding the kinetics of EV circulation and tissue penetration is crucial for optimizing their delivery to myocardial tissues.

### 5.11. Ethical and Safety Concerns

The clinical application of EVs raises several ethical and safety issues. The potential for EVs to transfer oncogenic material or induce immune responses necessitates thorough evaluation of their long-term effects. Off-target delivery and unintended cellular uptake could lead to adverse outcomes, including inflammation or fibrosis. Rigorous preclinical and clinical studies are essential to assess the safety profile of EV-based therapies and establish guidelines for their use in regenerative cardiology. In parallel, regulatory frameworks established by agencies such as the FDA and EMA provide guidance on EV characterization, safety evaluation, and clinical trial design, ensuring that ethical and biosafety considerations are rigorously addressed [57,58].

Addressing these challenges requires interdisciplinary approaches combining molecular biology, bioengineering, and clinical expertise. Advancements in EV isolation techniques, cargo engineering, and in vivo tracking methods are pivotal to overcoming these barriers. Furthermore, comprehensive safety evaluations and ethical considerations must guide the development of EV-based mitochondrial therapies to ensure their efficacy and patient safety [59,60]. Table 3 summarizes the major technical, biological, and ethical challenges of EV-mediated mitochondrial modulation alongside proposed strategies to address them.

## 6. Future Directions

Advancing the clinical application of extracellular vesicle (EV)-mediated mitochondrial modulation in regenerative cardiology necessitates a multifaceted, cross-disciplinary approach, integrating therapeutic strategies, standardization efforts, and cross-disciplinary collaboration.

Therapeutic Roadmap

**Figure 3:**
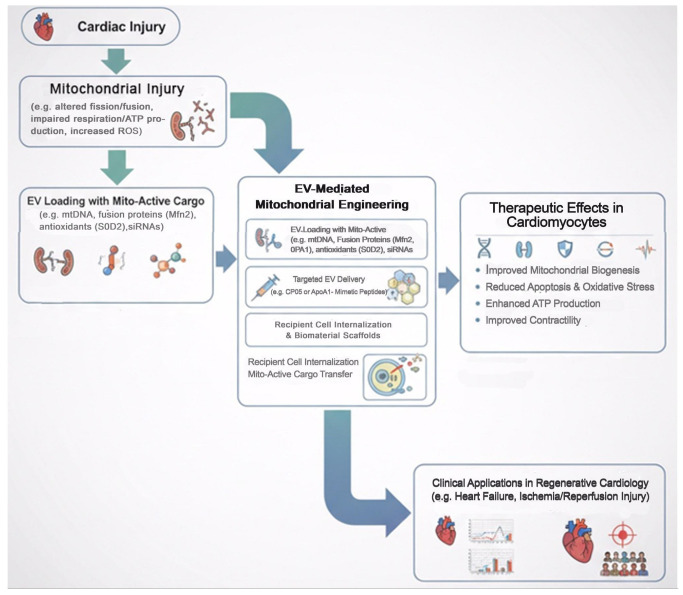
Therapeutic Roadmap: From EV-Mediated Mitochondrial Engineering to Clinical Applications.

To transition from preclinical models to clinical settings, a structured roadmap is essential. This includes optimizing EV isolation and characterization protocols to ensure reproducibility, scalability, and batch-to-batch consistency. Development of targeted EVs with enhanced mitochondrial delivery capabilities is critical, and strategies combining EVs with small molecules or immunomodulators may further potentiate therapeutic efficacy. Clinical trials should evaluate safety, efficacy, optimal dosing, and long-term outcomes, including potential adverse effects (Figure 3).

### 6.1. Clinical Trial Readiness and Regulatory Frameworks

A prerequisite for clinical translation is the establishment of robust manufacturing and regulatory frameworks. GMP-grade EV production has been demonstrated using bioreactor-based expansion of MSCs and iPSCs, coupled with scalable isolation techniques such as tangential flow filtration and size-exclusion chromatography [57,58,61,62]. Regulatory agencies (FDA, EMA) emphasize standardization of characterization metrics (particle number, protein content, potency assays), long-term safety evaluation, and reproducibility across batches [63].

For mitochondrial-targeted EVs specifically, additional checkpoints are required: biodistribution studies, mitochondrial cargo quantification, and assessment of off-target effects, including potential transfer of oncogenic or pro-inflammatory mtDNA. Several early-phase clinical studies using EVs for cardiac or neurological indications provide proof-of-concept for safety and feasibility [64,65], yet no trial has explicitly focused on EV-mediated mitochondrial repair. Building this translational bridge requires integrated pipelines combining GMP production, validated mitochondrial potency assays, and regulatory harmonization across agencies, enabling safe and reproducible clinical translation.

### 6.2. Cross-Sector Translational Roadmap

A structured roadmap bridging academia, biotech, and pharma is essential to accelerate adoption of EV-mediated mitochondrial therapies. Key elements include:

- GMP-grade production: scalable bioreactor expansion and standardized isolation.
- Potency assays: designed for mitochondrial rescue (bioenergetic restoration, uptake, functional readouts).
- Safety and regulatory checkpoints: biodistribution, immunogenicity, and long-term off-target effect monitoring.
- Integration with biotech/pharma pipelines: enhanced drug delivery, engineered EV cargo for mitochondrial modulation, and alignment with regulatory requirements.

Translational note: combinatorial strategies, such as EVs + small molecules or EVs + immunomodulators, may enhance repair efficiency and accelerate clinical impact. Collectively, these steps ensure reproducible, safe, and effective applications of mitochondrial-targeted EV therapies.

### 6.3. Personalized EV–Mitochondria Modulation

Patient-specific genotypes, including mtDNA variants and nuclear-encoded mitochondrial genes, should inform EV cargo selection. Tailored approaches aim to maximize efficacy and minimize variability in treatment response.

### 6.4. Standardization

Uniform protocols for EV preparation, characterization, and application are imperative. Standardization will facilitate cross-study comparisons and enhance reproducibility. International guidelines should harmonize methodologies, ensuring consistency and reliability in EV-based research and therapies.

### 6.5. Cross-Disciplinary Collaboration

The complexity of mitochondrial dynamics and EV biology necessitates collaboration across disciplines. Bioengineers can design EVs with specific targeting capabilities; systems biologists can model EV–mitochondrial interactions; cardiac surgeons and clinicians are critical to translating laboratory findings into clinically feasible interventions.

## 7. Conclusions

Mitochondrial dynamics—coordinated cycles of fusion, fission, mitophagy, and biogenesis—remain central to cardiomyocyte bioenergetics, stress adaptation, and structural homeostasis. Preclinical evidence consistently demonstrates that extracellular vesicles (EVs) can modulate these dynamics through dual mechanisms: (1) direct mitochondrial delivery via mitoEVs, which restore ΔΨm, ATP production, and respiratory chain integrity, and (2) regulatory modulation through cargoed miRNAs, mitochondrial proteins, and signaling molecules that influence DRP1 phosphorylation, MFN1/2 and OPA1 expression, and PINK1/Parkin-mediated mitophagy. These biomolecular interventions collectively reduce ROS accumulation, preserve calcium handling, limit apoptosis, and attenuate maladaptive remodeling following ischemia–reperfusion or chronic cardiac injury [7,16,24]

Engineered EVs, featuring targeted surface ligands, mitochondria-stabilizing proteins (e.g., SIRT3, ATP5A1), or miRNA cargo, enhance myocardial tropism and mitochondrial rescue efficiency, offering translationally tractable approaches for precision cardiotherapy [47,49]. Preclinical large-animal models validate the therapeutic impact of these strategies, showing improved ejection fraction, reduced infarct size, and bioenergetic reconstitution of injured myocardium.

Despite these advances, challenges persist. EV heterogeneity, lack of standardized isolation and quantification protocols, incomplete biodistribution tracking, and limited human trial data impede clinical translation. Addressing these gaps requires harmonized multi-omic characterization, quantitative functional assays, and rigorous safety evaluation, alongside integrative approaches that combine EV-mediated mitochondrial restoration with systems-level understanding of myocardial networks.

In summary, EV-mediated modulation of mitochondrial dynamics represents a mechanistically rich and clinically promising strategy in cardiac regeneration. By combining organelle replacement, network-level signaling, and bioenergetic reprogramming, this approach provides a grounded, reproducible, and scalable framework for cardiac repair. Harmonized protocols, mechanistic elucidation, and translational readiness remain essential to advance this strategy from preclinical promise to clinical practice.

Moving forward, targeted preclinical studies should prioritize combinatorial EV strategies, dose–responsedose-response optimization, and early-phase clinical trials to validate safety, biodistribution, and functional efficacy, thereby accelerating the path toward human implementation.

Finally, coordinated preclinical-to-clinical pipelines that incorporate standardized potency assays for mitochondrial rescue (ΔΨm/OCR/ATP assays), GMP-scalable manufacture of EV subfractions (including mito-EVs), and prospective monitoring for immune responses to extracellular mitochondrial cargo will be essential to move EV-based mitochondrial therapies safely into human use.

Moving forward, targeted preclinical studies should prioritize combinatorial EV strategies, dose–response optimization, and early-phase clinical trials to validate safety, biodistribution, and functional efficacy, thereby accelerating the path toward human application.

Finally, coordinated preclinical-to-clinical pipelines that incorporate standardized potency assays for mitochondrial rescue (ΔΨm, OCR, and ATP analyses), GMP-compliant large-scale manufacture of EV subfractions (including mitoEVs), and prospective monitoring for immune responses to extracellular mitochondrial cargo will be essential to bring EV-based mitochondrial therapies safely and effectively into clinical use.

## Figures and Tables

**Table 1:** Key Molecular Players in EV-Mediated Mitochondrial Regulation.

No	Category	Player	Function/Regulation
1.	Mitochondrial Fusion [12,26,27]	MFN1/2 (Mitofusins)	Mediate outer-membrane fusion. Upregulated by EV-delivered miRNAs (miR-30 family)
		OPA1	Mediates inner-membrane fusion. Upregulated by EV-delivered miRNAs
2.	Mitochondrial Fission [12,13,28,29]	DRP1 (Dynamin-related protein 1)	Executes mitochondrial fission. Regulated by site-specific phosphorylation (Ser616 activation, Se 637 inhibition). Downregulated by EV-delivered miRNAs. Recruited by receptor adaptors.
		MFF, FIS1	Receptor adaptors that recruit DRP1 to the outer mitochondrial membrane
3.	Quality Control and Turnover [12,13]	PINK1 (PTEN-induced kinase 1) [30]	Stabilizes on depolarized mitochondria; activates Parkin.
		Parkin [31]	E3 ubiquitin ligase recruited by PINK1; leads to ubiquitination and autophagic clearance (mitophagy). Activated by EVs.
4.	Mitochondrial Biogenesis [2,12]	PGC-1α	Master regulator of mitochondrial biogenesis. Upregulated by EV-delivered miRNAs (miR-499, miR-30 family→ Reduce DRP1 activity, increased MFN1/2, OPA1. Proteins: SIRT3 → enhances mitochondrial function and reduces ROS)
5.	EV Cargo [3,14,15]	mtDNA, Mitochondrial Proteins (e.g., TFAM), Cardiolipin, Mitochondrial RNAs, miRNAs	Cargo delivered by EVs to recipient cardiomyocytes. miRNAs can regulate DRP1, MFN2, opa1 expression and post-translational modification.
6.	Functional Outcomes	ΔΨm (Mitochondrial Membrane Potential), ATP/ADP ratio, ROS, BAX/BCL-2, Caspase activation	Restoration of ΔΨm, improvement in ATP/ADP ratio, reduction in ROS, inhibition of apoptosis through modulation of BAX/BCL-2 balance and caspase activation.

Mitochondrial Dynamics and Components: ROS: Reactive Oxygen Species, ΔΨm: Mitochondrial Membrane Potential, MFN1/2: Mitofusin ½, OPA1: Optic Atrophy 1, DRP1: Dynamin-Related Protein 1, MFF: Mitochondrial Fission Factor, FIS1: Mitochondrial Fission 1 Protein, mtDNA: Mitochondrial DNA, TFAM: Mitochondrial Transcription Factor A, PINK1: PTEN-induced Kinase 1, PGC-1α: Peroxisome Proliferator-activated Receptor Gamma Coactivator 1-alpha.nSources: MSCs, iPSC-CMs, Cardiac Progenitors. Extracellular Vesicles and Related: EVs: Extracellular Vesicles, MDVs: Mitochondrial-Derived Vesicles, mitoEVs/M-EVs: Mitochondrial-derived Extracellular Vesicles (or Mitochondria-containing Extracellular Vesicles), MSC: Mesenchymal Stem Cells, iPSC: Induced Pluripotent Stem Cells. Integration point: targeting mitochondrial dynamics via EV delivery couples organelle replacement/repair (mitoEV transfer) with network-level reprogramming (miRNA/protein cargo), offering a dual mechanistic strategy to restore cardiomyocyte bioenergetics and limit maladaptive remodeling. Key translational challenges include EV heterogeneity, quantitative assays for mitochondrial functional rescue, biodistribution, and standardized isolation/characterization protocols [5,23,32].

**Table 2:** Trials evaluating EV-mediated mitochondrial effects in cardiac contexts (2019–2025).

No	Study (Year)	Model/Phase	EV Source/Delivery	Key Mitochondrial Endpoints/Findings	Major Translational Limitation(s)
1.	O’Brien et al., 2021 [35] CA, USA	MSC-derived Large-EVs (L-EVs) (preclinical from Phase 1 SENECA trial [42])	IPSC-CM injured with doxorubicin and then treated with mito-EV from MSC	Reduced infarct size, decreased inflammation, improved organ regeneration	Variability in EV sources and delivery methods (large EV and small EV gave different results); inconsistent clinical outcomes
2.	Sasaki et al., 2022 [43] Fukuoka, Japan	In vitro human models and in vivo murine models with acute muscle injury and chronic kidney disease (preclinical)	MITO-Porter-mediated mitochondrial delivery to cardiac progenitor cells; transplantation	Enhanced mitochondrial activation, improved cardiac function post-myocardial ischemia–reperfusion	Complex delivery system; potential immunogenicity
3.	Liu et al., 2024 [10] Shanghai, China	In vitro human models and in vivo murine models with myocardial ischemia/reperfusion (M/R) injury (Preclinical)	Cardiac-derived EVs; Intravenous injection	Delivered ATP5a1, improved mitochondrial function, protected against ischemia/reperfusion injury	Focused on cellular assays; in vivo efficacy needs further validation.
4.	Lou et al., 2025 (Skeletal muscle from isolated healthy tissues) [44] Chengdu, China	In vitro human models and in vivo murine models with acute muscle injury and chronic kidney disease (preclinical)	Autologous stem cell-derived mito-EVs; intramyocardial/intracoronary	Restored myocardial OCR, increased ATP, reduced infarct size; evidence of mitochondrial incorporation into host cardiomyocytes	Limit to small animal, Scalability of mito-EV isolation; long-term fate of transferred mitochondria
5.	NCT06002841 [41] Valle del Cauca, Colombia	Early-phase clinical (registered)	MSC-derived EVs, intravenous	Safety and feasibility endpoints planned (cardiac patient population)	Early-phase; dosing and potency assays not standardized in registry
5.	Nie et al., 2025 [45] China	In vivo murine MI models with cardiac fibrosis from MSC-derived mitoEVs (preclinical)	Hypoxia-preconditioned bone marrow-derived MSCs; systemic delivery	Reduced infarct size, attenuated cardiac fibrosis, improved left ventricular function	Limited to small animal with human applicability remain uncertain

**Table 3:** Major Challenges and Proposed Solutions in EV-Mediated Mitochondrial Modulation for Cardiac Regeneration.

Challenge	Proposed Solution
Inconsistent EV isolation and characterization	Standardized protocols (ultracentrifugation, SEC, TFF), multi-omic characterization [57]
EV tracking and biodistribution	Advanced imaging (super-resolution, in vivo labeling), CRISPR barcoding [58]
Quantifying mitochondrial effects	Molecular assays, bioenergetic measurements (OCR, ATP/ADP), functional readouts
Off-target delivery/immune response	Engineered EVs with cardiomyocyte-targeting ligands, immunomodulatory cargo
Long-term safety and ethical concerns	Rigorous preclinical studies, regulatory oversight, standardized reporting frameworks

## Data Availability

All data used in this review were extracted from peer-reviewed published articles. No additional unpublished datasets or statistical code were generated.
